# Atlanta Society of Medicine

**Published:** 1896-01

**Authors:** W. L. Champion

**Affiliations:** Secretary; Atlanta, Ga.


### *SOCIETY REPORTS.*

### ATLANTA SOCIETY OF MEDICINE.

ATLANTA, GA., December 3, 1895.

In the absence of both the President and Vice-President, DR. W. A. CROW was called to the chair.

DR. LONGINO had been appointed to read a paper on the "Etiology and Pathology of Diphtheria," but was absent, and on motion of DR. MCRAE, Dr. BELL was invited to take his place.

DR. BELL stated as follows: "I do not think there is anyone who entertains an idea that diphtheria is due to anything else than Klebs-Lœffler bacillus. There are some features about this bacillus that do not come under my head but which I will mention. They are somewhat startling to me. During my connection with the New York hospitals we had a diphtheria epidemic there, and we gave tests to throats not apparently diphtheritic and found a number of cases of typical Klebs-Lœffler bacilli in throats that were perfectly normal. Many of these cases went through without having diphtheria. Out of forty cases of typical Klebs-Lœffler we took twenty cases and used a mild antiseptic wash and none of these twenty cases developed diphtheria, and only two out of the other twenty developed diphtheria. It is a startling fact that out of forty cases of Klebs-Lœffler only two developed diphtheria. This is difficult to explain.

"The Pathology of Diphtheria is something that I really do not feel that I could discuss without preparation. I have found in autopsies of diphtheria that this membrane appears in the throat in laryngeal cases. In some cases we found that the membrane could be traced into tubes that were so small as to be completely obliterated by the membrane. Another fact that is peculiar and that I noted quite carefully in this epidemic, that there were children apparently perfectly well and with a temperature of 98½ or 99 and yet their throats would be affected, and also in other cases in which the temperature would suddenly run up and upon examining their throats would find the poison. We had a number of cases of the poison reaching into the lungs and which gave typical cases of pneumonia. The glands of the neck are involved in a number of these cases, and I have noted that an opinion has been formed that those cases where the glands in the neck become early involved that they are malignant. They nearly always proved to be and therefore almost all fatal. The cases that show this early form of malignancy seem to be of the type of tonsillar. It seems to show that absorption from the larynx is not so marked as from the tonsillar surface."

In the absence of DR. TODD, DR. J. W. DUNCAN was invited to discuss the "Symptoms of Diphtheria."

DR. DUNCAN stated: "Dr. Bell will excuse me for a remark or two on the etiology. My opinion is that the greater number of diphtheritic cases that we have are the result of drinking well water. It is unsafe to drink the well water of the city of Atlanta. I believe there are a great number of people drinking well water thinking it pure water. I do not think there is a pure well in the city.

"I have seen a good deal of diphtheria, and usually when you are called to a case and before you examine the throat you will notice a peculiar pulse, a weak and soft pulse. I do not think it is a local disease of the throat. After a few hours, and even before the temperature has gone up very high, you may see a membrane form on the tonsils, pharynx, uvula, and soft palate. Sometimes it forms in the nose at the same time and extends down into the larynx, trachea, and the larger bronchi. If you see it a few days after it sets in and it has involved the nasal cavity the child has great trouble in breathing. Again, the symptoms may not be so severe; the membrane may be only in the throat and the nose be free, and the child go on for several days and the temperature not run high, but later on other complications may arise. The kidneys may not act well and there will be albumen in the urine. The patient will gradually get worse as the kidneys do not act and often at this time you will have paralysis, which sometimes affects both arms and both legs. You may have a case which is apparently doing well, and for a number of days you think the patient is improving and the child will be able to sit up and notice its playmates, and all at once it will die of paralysis of the heart.

"The frequent, feeble, soft pulse continues throughout the course of the disease, unless in rare cases it becomes extremely slow from a weakened condition of the inhibitory nerves.

"I remember one case, a girl seven years of age, who had diphtheria involving the palate, uvulva, and bronchi, and later on she became paralyzed. She could not swallow and her arms or legs could not be used; she had to be nourished entirely by enemata. About the third week her pulse dropped down to thirty-eight and remained that way for a day and night. While being helped to the chamber she fainted. I was called in and I gave her a stimulant. Her pulse went up to ninety, and the next day went up to one hundred and ten. She gradually recovered and is now perfectly well, stout, and healthy. This was one of the worst cases I ever saw to recover.

"Other symptoms that I might mention; one is, if there is an abrasion on the skin of the body the membrane will form on that. I have known physicians to apply a fly plaster and make a blister and immediately upon its removal the membrane would form on this surface. I could not see any benefit from this. They seldom cough in diphtheria as much as the cause would indicate. There seems to be an obtunded condition of the nerves of the pharynx and the larynx. In the beginning of the disease they frequently vomit, as they do also in scarlet fever, but this is not a symptom of the disease. The bowels are constipated rather than otherwise. The membrane, whether anything is used to cause it to be thrown off or not, disappears in from seven to nine days. It may be thrown off and it will form again."

On the Pathology of Diptheria DR. F. W. MCRAE stated as follows: "I wish to preface my remarks by giving my individual experience. During eleven years in Atlanta I have seen six cases of diphtheria, three in consultation and three of my own, and I have not treated any of them. Of the three cases in which I was called into consultation, two were grown people; one a lady thirty or thirty-five years of age, and the other was Dr. Cowan. Both of these died. The other case was the child of Mr. Liebman, and this also died. The cases were treated with local applications and internal remedies. The other cases I have seen were very severe. I turned the cases over to Dr. Sommerfield and simply watched them. The results were remarkable. He used the antitoxin, and while it did not entirely free them from complications, they recovered.

"A very important thing to which the discussion should be directed is to the precautions the physicians should observe against spreading the disease themselves. We go into a room where there is a case of diphtheria and come out and wash our hands and go to the next family. I think some cases can be traced directly to physicians. I am of the opinion that the disease is due to the Klebs-Lœffler bacillus, and that it can be prevented from spreading by the use of proper precautions. I think that all the outer clothing of the physician should be changed, and everything that comes in contact with the child, the face, hands, and beard, should be washed with soap and then with a 1 to 1,000 bichloride solution, and that the room should be thoroughly fumigated and scoured with bichloride. By observing proper precautions I think it can be kept from spreading. Although all cases cannot be traced to direct contagion, still I think they can to a great extent. By tracing you will find cases which seem to have been given from animals to children and from children to cats and dogs and the disease spread in this way. I have seen reports of cases that seemed to have been traced to milk from a certain dairy.

"I believe in hygienic treatment. I do not think there is anything better than hygienic treatment with the use of antitoxin. I am a firm believer in antitoxin. I do not believe that it will cure all cases, as no drug or plan of treatment can accomplish this, for in cases where the system is in low condition the disease often goes so fast that no treatment will amount to anything. I find in looking up, that, with a few exceptions, where antitoxin has been used scientifically it has been successful. It has been used in the London hospitals and has been largely used on the continent and in New York, and the reports are most favorable. From the best information that I can gather from all sources the mortality has been reduced from forty or fifty per cent. down to eighteen per cent. I do not think that all antitoxin is good, as some of it does not contain the specific toxin at all, and therefore is valueless. Aronson's antitoxin seems to be the best, and the dose is about five cubic centimetres, and should be given with the strictest antiseptic precautions and in the interscapular region. In the cases mentioned, I watched them from four to twelve hours before turning them over to Dr. Sommerfield, and the membrane had formed and the temperature was one hundred and three. In twenty hours from the injection of the antitoxin the temperature was less than one hundred, and in thirty-six hours in both cases the membane had practically disappeared and one of the cases never had any unpleasant symptoms afterward. The other had some kidney complications. I wish to state from my practical observation and from my reading that I am very strongly impressed with the value of antitoxin in the treatment of diphtheria. It is along the line in which we may expect a great deal in the future. I think that the ordinary treatment is entirely insufficient; that the use of drugs internally, other than supportive, is simply palliative. For internal and local effect I should cling to the old prescription of chlorate of potash, glycerine, and iron. I believe that diphtheria is primarily a local trouble. I think it is important to sustain the patient with good nourishments and stimulants, whatever plan of treatment is adopted. Where antitoxin is used I would advocate the spraying of the throat with peroxide of hydrogen. Of the operative treatment I am not competent to speak, as I have not operated myself, and the cases I have seen which required it died. Other cases which I have seen, or started to see, were dead when I got there. So that, in all, I have eight cases of diphtheria; two of them died without my seeing them, and three died and three got well. I think that Dr. Duncan is eminently correct in his statement that blisters are not indicated, as this simply makes a wound and increases the danger. The point I wish to emphasize is the proper use of antitoxine. If my own child had diphtheria and I were to treat it without antitoxin I would feel culpable. I believe it ought to be used. I do not think the claims are sustained that it increases kidney complications. You have the complications with or without antitoxin, but I believe it materially lessens them."

On the after effects of diphtheria DR. C. C. STOCKARD stated as follows: "A source of great anxiety to the physician, and of danger to the patient who has passed through an attack of diphtheria, is a form of paralysis which occurs, according to different authors, in a large per cent. of all cases. It occurs much more frequently in some epidemics than in others, and varies from a slight paresis of a few muscles to an extensive and oftentimes fatal paralysis, and this accounts probably for the variation in frequency stated by different authors. It occurs more frequently, in proportion to the number of cases, in older children and adults than in young children, but according to the best authorities is not more likely to occur after a severe than after a mild attack of diphtheria. The pathological condition is one of peripheral neuritis, beginning almost always at the point of local manifestation of the disease. In the light of bacteriology there is little doubt but that the cause is ptomaïne poisoning. The approach of paralysis is ordinarily shown by a nasal tone of voice, and regurgitation of fluid through the nares, though absence of knee jerk, it is claimed, exists prior to these. I do not feel disposed to attach very much importance to this, however, as knee jerk is much less marked and more difficult to bring out in small children. On examination of the throat, the soft palate, velum, and uvula are found relaxed and swinging back and forth with the respiration. In some cases only one side is affected, and when both are, it is more marked on one side. With motor paralysis there is generally some loss of sensibility. Fortunately in many cases this condition lasts a few weeks and passes off, while in others, however, it is followed by paralysis of accommodation, disturbing vision, of the lower limbs, and finally the upper extremity, trunk, viscera, and sphincters. A guarded prognosis must always be given, as death is liable to occur suddenly from paralysis of the heart or diaphragm, or from the passage of food into the respiratory tubes. Leaving out these cases, recovery usually occurs slowly, the restoration of function being in the same order as the attack. Paralysis following diphtheria being the result of diphtheria toxines, if the antitoxin treatment accomplishes what its advocates claim, will very rarely occur when this treatment comes into universal use. When it does occur, the strength of the patient must be sustained by careful feeding, through the tube should it be necessary on account of inability to swallow; tonics, such as strychnia, arsenic, iron and quinine. The heart must be carefully watched and failure here guarded against. Electricity is beneficial as a general tonic, as well as upon the local nervous manifestations."

In the discussion DR. CROW stated: "I have been very much interested and have nothing to add to what has been said. I have had very little practical experience with diphtheria and have not seen a case for the last three or four years that I can recall. During my recent visit abroad I made inquiry in regard to the treatment of diphtheria in the use of antitoxin, and found they were using the antitoxin a great deal, although in Edinborough they have abandoned the use of antitoxin and have resorted to the old treatment. I do not favor the removal of the membrane, which was in vogue several years since, as it leaves a raw surface. I did not see any cases on the continent, but learned that they were using the antitoxin and were much pleased with it, as they got better results from this than from any other form of treatment. The French are highly in favor of not only antitoxin, but all treatments of this kind. In the Middlesex hospital of London I saw fifteen or twenty cases of diphtheria in going through the wards, and in these cases they were using the antitoxin, and it was highly spoken of. In Dublin it is used by some, while others do not favor it. I would certainly give antitoxin a trial if any of my family had diphtheria, or I was called to a case that was diphtheritic."

In the discussion DR. BELL spoke as follows: "As to the primary infection of diphtheria, I do not think there is any question about the fact that it is a local trouble. There are cases in which one would be misled as to the fact of its being a systemic trouble by having enlargement of the glands and only a small point of the membrane. This would cause one to come to the conclusion that this person was poisoned before the membrane came into the throat. The glands were simply infected before the membrane was sufficient to be seen by the eye at all. I think this is the explanation to the fact that some claim that the trouble is a systemic trouble. I think that the greater number who have handled cases will take the point that it is primarily local. It has not been fully established that antitoxin is of a great deal of virtue, but I would certainly not let a case go without using the antitoxin. While [mortality has been reduced since the introduction of antitoxin, yet there are other things that come into consideration, and I think it well to consider these, although I am an antitoxin man, and have used it with most excellent effect. In the treatment of diphtheria the chlorate of potash is reliable, but I would be afraid to use it where the kidneys are diseased."

DR. DUNCAN stated: "As regards to diphtheria being a local trouble, it is a local trouble only for a few hours. I am satisfied that if you are called to a case of diphtheria a few hours after they state they are unwell, you will notice a peculiar appearance, and you may examine the throat as carefully as you please, and you can find no membrane or sign of a membrane whatever. The patient will have the membrane from five to seven hours later. If it is simply a local trouble, it seems to me that it could be controlled by the use of peroxide of hydrogen, or something of this kind. I am glad to hear Dr. McRae speak so favorably of the antitoxin. I have read considerable about it in the journals, and I did not have a favorable opinion of it. I have been in three epidemics of diphtheria, and in about twenty per cent. of the cases the membrane extended into the nose. I do not believe in appplying a strong acid to the membrane, or removing the membrane by force. I doubt whether we ought to use the bichloride solution in strong quantities. As regards the use of chlorate of potash, I would not hesitate to give a child one or two grains every two or three hours. It is not only curative, but the best preventive I know of. I have gone to families where there were seven in the family, and put the well ones on the iron and bichloride treatment, and they would have no trouble. In one family one child refused to take the preventive, and it had the disease. I cannot recall a case where the iron and chlorate of potash were used and the disease developed afterward. I have tried it in a number of families where the families were together, and had no trouble. I also give milk punches and brandy, and do not allow them any rich food at all. Another thing that has a good effect, is pilocarpine in small doses; just enough to increase the flow of saliva. It helps to loosen the membrane. I have used other tonics a great deal, but I believe some stimulation is necessary. I have also used a number of local applications on the throat, but I think that a poultice of salt saturated with water is about as good as anything else, as it lessens the trouble in breathing."

DR. McRAE stated : "I do not care to add much to what I previously said, except that while I did not say stimulants, I mentioned supportive treatment and liquid diet, which are most essential whatever treatment is adopted. I neglected to mention the immunization by the use of antitoxin. If properly administered, I think it will prevent it. I do not think there is any danger in the use of chlorate of potash guardedly, even where there is albumen in the urine. I think it is of value in relieving the catarrhal condition of the throat. I do not think any treatment should be directed toward the removal of the membrane *per se*, as I do not think the membrane is doing any harm, unless in the larnyx, and therefore its removal would not do any good, and if forcibly removed it leaves a raw surface. I should be very much afraid to use pilocarpine, as it would tend to induce sloughing. I do not wish to appear dogmatic in the advocacy of antitoxin treatment, but I am very much in favor of it. I know there are two sides, and advocates on both sides, but I am firmly convinced that the weight of competent opinion is in favor of antitoxin. When a remedy of this character is introduced, it is lauded and condemned until it finds its proper place, and I believe it will find a place, and be recognized as both curative and prophylactic."

DR. STOCKARD: "I am a firm believer in antitoxin, and I think that in the hospitals wherever used much, it has been advocated. I think that the opposition to it in some of the English hospitals is due mainly to the fact that it was first introduced by a Frenchman. The investigations in the hospitals in New Orleans have been extensive, and they have been convinced of the efficacy of the treatment. I think it is the duty of the physician to give antitoxin to every child that has been exposed. I think that it will take the place of everything else. When Dr. Duncan spoke of pilocarpine, I did not know it had been used in diphtheria for twenty years, but I have seen where it was spoken of very highly in connection with diphtheria."

W. L. CHAMPION, M. D., Secretary.

